# Role of mTOR Signaling in Tumor Microenvironment: An Overview

**DOI:** 10.3390/ijms19082453

**Published:** 2018-08-19

**Authors:** Fabiana Conciatori, Chiara Bazzichetto, Italia Falcone, Sara Pilotto, Emilio Bria, Francesco Cognetti, Michele Milella, Ludovica Ciuffreda

**Affiliations:** 1 Medical Oncology 1, IRCCS Regina Elena National Cancer Institute, Rome 00144, Italy; fabiana.conciatori@ifo.gov.it (F.C.); chiara.bazzichetto@ifo.gov.it (C.B.); italia.falcone@ifo.gov.it (I.F.); francesco.cognetti@ifo.gov.it (F.C.); michele.milella@ifo.gov.it (M.M.); 2 Department of Molecular Medicine, University of Rome, La Sapienza, Rome 00185, Italy; 3 Department Medical Oncology Unit, Azienda Ospedaliera Universitaria Integrata, University of Verona, Verona 37100, Italy; sara.pilotto.85@gmail.com; 4 Medical Oncology, Fondazione Policlinico Universitario “A. Gemelli” IRCCS Università Cattolica del Sacro Cuore, Rome 00168, Italy; emilio.bria@unicat.it

**Keywords:** mTOR, tumor microenvironment, angiogenesis, immunotherapy

## Abstract

The mammalian target of rapamycin (mTOR) pathway regulates major processes by integrating a variety of exogenous cues, including diverse environmental inputs in the tumor microenvironment (TME). In recent years, it has been well recognized that cancer cells co-exist and co-evolve with their TME, which is often involved in drug resistance. The mTOR pathway modulates the interactions between the stroma and the tumor, thereby affecting both the tumor immunity and angiogenesis. The activation of mTOR signaling is associated with these pro-oncogenic cellular processes, making mTOR a promising target for new combination therapies. This review highlights the role of mTOR signaling in the characterization and the activity of the TME’s elements and their implications in cancer immunotherapy.

## 1. Introduction

The mammalian target of rapamycin (mTOR) forms two functionally and structurally distinct multi-component complexes, named mTOR complex 1 (mTORC1) and mTOR complex 2 (mTORC2). These two complexes regulate several physiological processes, such as protein synthesis, biosynthesis of macromolecules, cytoskeleton remodeling, angiogenesis, homeostasis, survival, metabolism, autophagy, and response to stress [1]. Because of its key role in cell growth and differentiation, its deregulation is implicated in pathological conditions including neoplastic transformation and progression, such as in breast, gastrointestinal, liver, and prostate cancers [2].

Moreover, the mTOR pathway is involved in the differentiation, function, and metabolic regulation of adaptive/innate immune cells, as demonstrated by the use of rapamycin in clinical practice as an immune suppressant in organ transplant patients. Indeed, mTOR may regulate the activity of immune cells, such as macrophages and T cells, by regulating the expression of the inflammatory factors, such as cytokines/chemokines (i.e., interleukin (IL)-10, transforming growth factor (TGF)-β) and/or membrane receptors (i.e., Cytotoxic T-Lymphocyte protein 4 (CTLA-4) and Programmed Death 1 (PD-1)) [3]. The immune cells, recruited in the tumor microenvironment (TME) by the cytokines/chemokines-cytokines/chemokines receptor interactions, could exert the anti-tumor functions or promote cancer cells’ growth. Thus, inflammation plays a central role in the tumor dynamic and represents one of the hallmarks of cancer [4].

Along with the immune system, tumor vasculature is a key component of TME and can influence the tumor behavior and drug treatment; mTOR is involved in the regulation of tumor-related vascular formation, through the promotion of angiogenesis [5]. One of the most prominent effects of mTOR under a hypoxic condition is the translation of hypoxia-inducible factor (HIF) 1-2. The HIF transcription factors lead to the expression of the hypoxic stress response genes, including angiogenic growth factors such as vascular endothelial growth factor (VEGF), TGF-α, and platelet-derived growth factor β (PDGF-β) [6].

The mTOR-mediated cellular metabolism is implicated in the cancer cells’–TME interactions during the tumor progression and drug resistance, suggesting that phosphoinositide 3-kinase (PI3K)-/protein kinase B (AKT)-/mTOR-blockade may have the dual benefit of reducing the cells’ proliferation, migration, and survival, and enhancing the tumor immunosurveillance through both the downregulation of the immunosuppressive pathways and the activation of anti-tumor immune activities.

In this review, we describe the role of mTOR in tumor and non-tumor cells in order to better analyze the mechanisms of cancer progression and metastasis as well as drug resistance development.

## 2. mTOR Signaling

**Figure 1:**
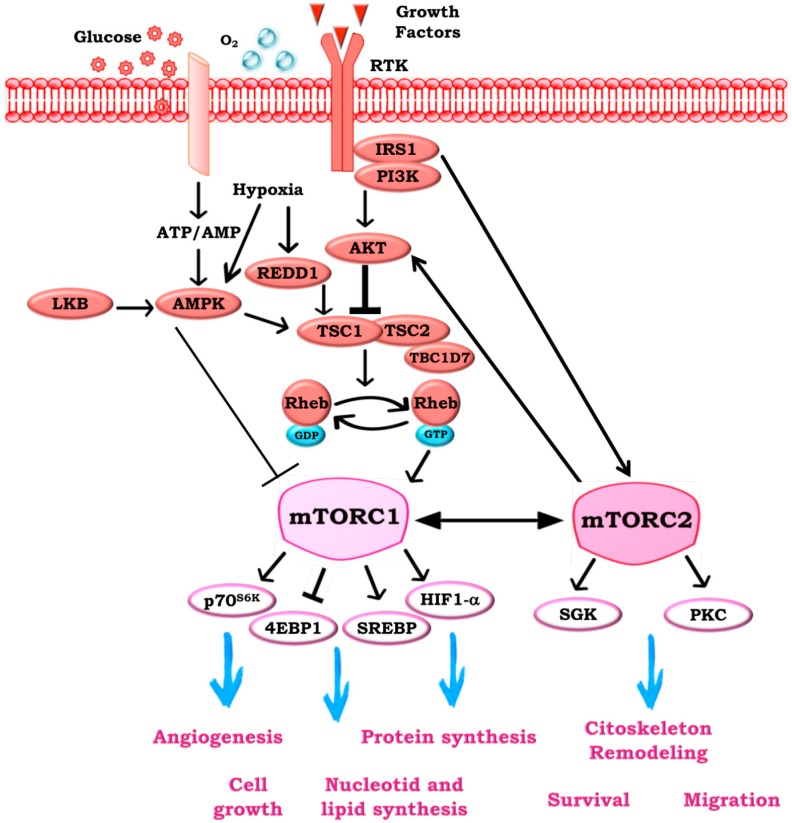
The mammalian target of rapamycin (mTOR) pathway. mTOR signaling is activated by extracellular signals like growth factors, nutrient, and oxygen levels via the phosphoinositide 3-kinase (PI3K)/protein kinase B (AKT) pathways. Extracellular signals may both inhibit the tuberous sclerosis complexes 1–2 (TSC1–2) to promote the accumulation of RAS homolog enriched in brain (Rheb)-GTP and the subsequent activation of mTOR complex 1 (mTORC1), and activate TSC1–2 complex to block mTORC1 by Rheb. Activation of 5′-AMP activated protein kinase β (AMPK-β) by low levels of energy results in direct phosphorylation and activation of the TSC1–2 complex. mTOR complex 1 (mTORC1) activation leads to the phosphorylation and activation of mTORC1 effector proteins ribosomal protein S6 kinase (p70*^S6K1^*) and 4E-Binding Protein 1 (4EBP-1), thus resulting in initiation of specific cap-dependent translation events. Then, mTORC1 regulates cell growth and protein translation through p70*^S6K1^* and 4EBP-1, as well as lipid synthesis through SREBP1, while angiogenesis through hypoxia-inducible factor (HIF)-1. The function and activation of mTORC2 is less well understood. It is thought to be activated by growth factors through the PI3K pathway and mTORC1. mTORC2 influences the cytoskeletal organization survival and migration.

mTOR is an evolutionarily conserved serine/threonine kinase belonging to the PI3K-related kinase family, which integrates a variety of exogenous cues to coordinate several cellular processes, including cell growth and metabolism (Figure 1) [2,7,8]. 

mTOR forms two functional multiprotein complexes, mTORC1 and mTORC2, which are characterized by the different binding partners that confer distinguishing functions upon them. mTORC1 includes mTOR, raptor, proline-rich AKT substrate 40 (PRAS40), DEP domain-containing mTOR interacting protein (DEPTOR), mammalian lethal with sec13 protein 8 (mLST8), Rac1, GRp58, Tel2-interacting protein 1 (Tti1) and Telomere maintenance 2 (Tel2), while mTORC2 includes rictor, mammalian stress-activated protein kinase interacting protein 1 (mSIN1), protein observed with rictor (Protor) 1/2, proline-rich protein 5 (PRR5), and heat shock protein 70 (Hsp70), in addition to mTOR, DEPTOR, mLST8, Rac1, GRp58, and Tti1 and Tel2 [1]. In both complexes, the mTOR kinase acts as the central catalytic component, whereas the scaffolding protein mLST8, the regulatory subunit DEPTOR, and Tti1/Tel2 act as important regulators of assembly and stability. Furthermore, each complex is composed of specific components that contribute to complex regulation, substrate specificity, and subcellular localization [1].

mTORC1 is rapamycin-sensitive and its main targets are the proteins involved in mRNA translation, including the p70*^S6K1^* and 4EBP-1. Conversely, mTORC2 is insensitive to rapamycin and the promotes phosphorylation of the hydrophobic motif of protein kinase B (AKT), serum and glucocorticoid kinase (SGK), and protein kinase C (PKC) [9]. The mTORC1 activity depends on diverse positive signals, such as energy levels, oxygen, amino acids, or growth factors, and regulates several processes required for cell growth and metabolism, including ribosomal biogenesis, protein translation, and autophagy. mTORC2 has been characterized as a downstream effector of the insulin/ insulin growth factor (IGF)-1 signaling pathway and it is involved in the regulation of proliferation, survival, cytoskeletal remodeling, and cell migration [9,10,11]. In response to the insulin or growth factors, mTORC1 is mainly activated by PI3K/AKT signaling [12]; similarly, PI3K is also a key modulator of mTORC2, by promoting the binding of mTORC2 to ribosome [13]. PI3K is activated by receptor tyrosine kinases, G protein-coupled receptors, and RAS, and it acts as a kinase on the lipid second messenger phosphatidylinositol 4,5-bisphosphate (PIP2) to produce phosphatidylinositol 3,4,5-trisphosphate (PIP3), which recruits the phosphoinositide-dependent kinase-1 (PDK1) and AKT to the plasma membrane and thus activating the mTOR signaling [14]. Phosphatase and tensin homolog on chromosome 10 (PTEN) is a classical tumor suppressor and it acts as a phosphatase, by dephosphorylating PIP3 to PIP2 and thus reversing the action of PI3K and its downstream functions [15,16].

Because of the key role of mTOR signaling in regulating these fundamental biological processes, the deregulation of the PI3K-AKT-mTOR pathway is tightly connected to cancer initiation and progression, and several biological investigations have focused on targeting this pathway in cancer cells, also within therapeutic combinations [1]. Interestingly, recent evidence shows that treatment with PI3K-AKT-mTOR signaling inhibitors not only affects the tumor progression, but also tumor immunosurveillance within the TME [17].

## 3. Tumor Microenvironment

**Figure 2:**
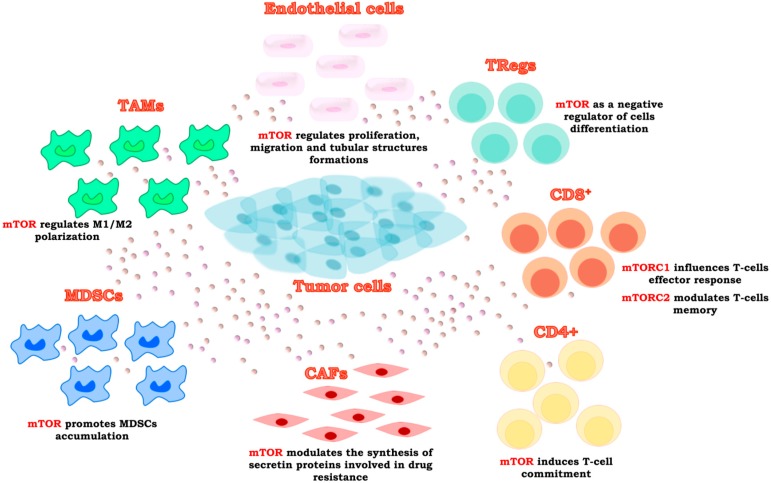
mTOR in characterization and the activity of the tumor microenvironment (TME) elements. mTOR signaling is involved in the modulation of several environmental inputs in TME, mainly composed by regulatory T cells (Treg), CD8^+^ and CD4^+^ lympohocytes, myeloid-derived suppressor cells (MDSCs), tumor-associated macrophages (TAMs), endothelial cells, and fibroblasts.

TME is composed of the stroma and its components, different cells types, and paracrine factors (Figure 2) [18]. Mounting evidence suggests the involvement of TME in cancer progression and drug resistance; indeed, in physiological conditions, the stroma acts as a physical barrier, whereas, during carcinogenesis, the presence of tumor cells induce changes that convert the adjacent TME into a pathological entity [18,19].

Several cell types contribute to the characterization of TME, including cancer and non-cancer cells [20]. Non-malignant components consist of cancer-associated fibroblasts (CAF), myeloid-derived suppressor cells (MDSCs), tumor-associated macrophages (TAM), and regulatory T cells (Treg); they all have a dynamic and tumor-promoting function during the carcinogenesis process [21,22]. CAFs are the major components of cancer stroma and promote tumorigenesis by both remodeling the extracellular matrix (ECM) and secreting cytokines [20]. The MDSCs are myeloid cells, involved in the inhibition of immune cells by releasing IL-10 and in the polarization of TAM towards a tumor-promoting phenotype [20]. Indeed, the TAMs usually display pro-tumorigenic properties and they are the major contributor to tumor angiogenesis [23]. The TAMs represent the prominent leukocytic infiltrate component in cancers and can comprise up to 50% of the cell tumor mass [24]. The cytokines in the TME directly regulate the phenotype switching of macrophages into M1 and M2 polarization, thus leading to the acquisition of distinct functional features; the M1 macrophages predominantly secrete pro-inflammatory mediators, whereas the M2 secrete the anti-inflammatory ones [25]. Numerous evidence suggest that infiltrating the macrophages switches their phenotype from the anti-tumoral M1 to pro-tumoral M2 during cancer progression, even if the TAMs are the unique polarized macrophages that express both M1 and M2 marker genes [25]. Tregs exert their suppressive activity via cell-to-cell contact (i.e., PD-1 and CTLA-4) or via the expression of soluble factors, such as TGF-β and IL-10 [26].

The ECM consists not only of a physical scaffold for TAM cells (fibrous and matricellular proteins and glycosaminoglycans), but also of the growth factors, cytokines, and hormones secreted by the stromal and tumor cells. ECM is characterized by biochemical properties specific for each tissue, and may be involved in overcoming the host’s immune surveillance [18,27,28,29]. 

In TME, the complex and dynamic network of cytokines, chemokines, and growth factors drive the inter- and intra-cellular communication that may modulate tumor/stroma interaction, including immune responses; indeed, the chemokines–chemokine receptors interactions recruit different immune cells into the TME and they regulate the tumor immune responses in a spatiotemporal regulated manner [30]. 

The cytokines are low-molecular-weight proteins, released by cancer, immune cells, and stromal cells, such as fibroblasts and endothelial cells, in order to regulate the cell proliferation, survival, migration, and death [31]. The chemokines are a type of cytokine with chemo-attractant properties and are divided in four subfamilies (C, CC, CXC, and CX3C), based on their primary structure and function; all have the main cysteine residues in their N-terminal regions [32]. The data obtained in our laboratories have suggested that the hyperactivation of the PI3K-AKT-mTOR pathway is involved in the up-regulation of specific cyto- and chemo-kines expression (i.e., IL-8 and VEGF), thus directly affecting the TME components [33,34]. De la Iglesia, et al. demonstrated that in PTEN-loss glioblastoma, the unphosphorylated signal transducer and activator of Signal transducer and activator of transcription 3 (STAT3), which transcriptionally represses IL-8, does not bind the IL-8 promoter, thus leading to an increased transcription and expression of IL-8 gene; in this way, IL-8 promotes the glioblastoma cell proliferation and invasiveness only in a genetic PTEN-loss context [35]. This relationship between PTEN-loss and a selective upregulation of IL-8 signaling has also been demonstrated in prostate carcinoma [36].

## 4. Immunoregulatory Functions of mTOR

Recent studies have established an important role for mTOR in the modulation of both innate and adaptive immunity, integrating different environmental inputs within the TME. Indeed, mTOR is involved in the regulation of many immune cellular functions promoting differentiation, activation of T cells, TAMs, and antigen-presenting cells (Figure 2) [37,38]. The effects of the mTOR complexes’ activation in the TME elements are summarized in Table 1.

It is actually known that the PI3K/mTOR inhibitors have an important immunomodulatory impact on the tumor microenvironment and angiogenesis. The modulation in the number and/or function of the specific TME cells involved in tumor progression is often associated with a better outcome in cancer therapy, and for this reason, the selective inhibition of the PI3K/mTOR axis correlates not only with the efficacy in leukemias, but also improves the immunotherapy in different solid cancer [27,55]. Recent studies showed that the inhibition of different hub of PI3K/mTOR pathways impacts the different TME components (Table 2).

### 4.1. T Lymphocytes 

Emerging evidence highlights a central role for mTOR in bridging the immune signals and metabolic cues to direct lymphocyte proliferation, differentiation, and survival [65].

The lymphocyte activation increases protein, nucleotide, and lipid biosynthesis and utilizes aerobic glycolysis to generate ATP during the rapid proliferation [40,66]. The metabolic programs regulated by mTORC1 make it an important link between metabolism and immune function [40,49].

#### 4.1.1. CD8^+^

CD8^+^ cytotoxic T cells, derived from naïve CD8^+^ T cells, are the major antitumor mechanism of the immune system because of their ability to target and kill cancer cells and maintain a memory response. Recently, mTOR has been identified as a regulator of memory CD8^+^ T cells differentiation [67]. It has been demonstrated that rapamycin modulates the CD8^+^ T cells induced by viral infection, showing that mTOR regulates the memory CD8^+^ T cells differentiation [68]. Indeed, it has been reported that mTORC1 negatively modulates the memory T cells formation [40].

Moreover, Pollizzi et al. [39] have demonstrated that mTORC1 and mTORC2 may play a different role in the regulation of CD8^+^ cells; mTORC1 positively influences the CD8^+^ T cells effector responses and glycolytic phenotype, while the mTORC2 activity is involved in the CD8^+^ T cells memory up-regulation. Moreover, the direct modulation of the mTOR-mediated lipid metabolism by the inactivation of the sterol regulatory element binding proteins (SREBP) pathway inhibits CD8^+^ T-cell proliferation in vitro [69].

#### 4.1.2. CD4^+^

The activation of naïve T cells can result in the simultaneous expression of the tumor specific antigens, Th (T helper) 1, 17, and 2, and it is now clear that mTORC1 and mTORC2 promote T cells commitment [42]. Indeed, Delgoffe and collaborators demonstrated that the mTOR-deficient CD4^+^ T cells failed to differentiate into Th1, Th17, or Th2 effector cells in vitro or in vivo; the loss of Rheb, an upstream activator of mTORC1, inhibits the differentiation of both the Th1 and Th17 cells, whereas the Th2 cells differentiation requires mTORC1 activation but not Rheb [41,42]. The loss of Raptor induces Th17 cells differentiation [70]. According to the key role of mTOR in Th differentiation, Templeton and collaborators have demonstrated that treatment with everolimus induces a statistically significant reduction in the numbers of CD4^+^ and an increase in the Treg population, in a dose dependent manner in metastatic prostate cancer patients, with an increase in progression free survival [58].

Recent studies have shown that antigen stimulation causes T cells to transit from catabolism to anabolism, and mTOR regulates this process by enhancing the T cells metabolic activity [71]. In antigen-stimulated T cells, the mTOR activates the glycolytic program inducing the expression of MYC and HIF-1α, which in turn mediates the expression of glycolytic enzymes and transporters [72,73].

#### 4.1.3. Treg

The forkhead box 3^+^ (FOXO3^+^) Tregs suppress inflammation and have an important role in tumor immunity through their role as a suppressor of the effector T cells. High numbers of Treg and a reduction of the CD8^+^ numbers in the tumor infiltrate are associated with a poor prognosis [40]. The reduction of mTOR signaling induces Treg expansion through the FOXO3 expression; in fact, as opposed to the CD4^+^ T cells, the mTOR axis regulates the lineage commitment between effector and regulatory T cells through the STAT transcription factor activation [41,74]. Despite the well-known role of the PI3K-AKTpathway in T-cell proliferation, the AKT and PI3K blockade selectively inhibits the Tregs’ proliferation with minimal effect on the other T cells’ population (CD4^+^ and CD8^+^). Indeed, the in vitro and in vivo studies display that the Tregs have an increased dependence on the PI3K-AKT signaling pathway, as compared to the other T cells [59].

Interestingly, mTOR may mediate different Treg functions, as demonstrated by the pharmacological and genetic modulation of the mTOR network. Indeed, the reduction of mTORC1 activity, by the deletion of Raptor, reduces the Treg function, and conversely, the over-activation of mTORC1 reduces the FOXO3 expression and converts Treg into effettor-like T cells, in TSC1-deficient models [44]. Moreover, the activation of mTORC2 in the PTEN-loss Tregs induces a reduction in their stability and their ability to differentiate [45,46]. It has been also demonstrated that the programming of the Treg suppressive functions is dependent on mTORC1; indeed, Zeng et al. have demonstrated that mTORC1 acts as a link between the immunological signals via the T cell receptor (TCR) and IL-2 to lipogenic pathways [75].

Recently, it has been reported that mTOR also modulates the role of PD-L1 as a regulator of the development, maintenance, and function of the induced regulatory T cells [76]. PD-1 is highly expressed on Treg cells and PD-L1 is widely expressed in several stroma non-hematopoietic cells and in various tumors; Lastwika and collaborators have shown that the activation of the mTOR pathway regulates the PD-L1 expression in vitro and in vivo in lung carcinoma [77,78].

### 4.2. TAMs

TAMs are a class of immune cells that are present in high numbers in the TME and are recruited by soluble factors (cytokines and/or chemokines) or derived from tissue-resident macrophages. TAMs are generally classified into M1 and M2, depending on their polarization; M1 TAMs are involved in phagocyte-dependent inflammation and in antitumor response, whereas M2 TAMs inhibit the phagocytic function thereby being more tolerant towards tumor growth. However, it has been demonstrated that this is a simplification of the TAM’s classification; Qian et al. showed that TAMs can share both M1 and M2 phenotype and functions, thus resulting in a difficult interpretation of their role in TME [40,79,80]. 

Early evidence highlights the role of mTOR signaling in macrophage activation and differentiation. Indeed, Byles et al. showed that the loss of TSC1 activity in the macrophage leads to constitutive mTORC1 activation, and the consequent decrease in IL-4 production induces M2 polarization [47]. Consistent with these results, different studies demonstrated that the mTORC1 downregulation by different pharmacological and genetic approaches results in both decreased proinflammatory cytokine production by macrophages, with a consequent reduction of inflammation, and unbalances in macrophages M1 polarization [48,81]. Moreover, the role of the mTOR pathway in macrophage activity is involved not only in M1/M2 polarization, but also in processes, such as autophagy, which could indirectly influence the outcome of tumor progression. Indeed, Shan and collaborators demonstrated that rapamycin both reduces the M2 macrophage polarization by down-regulating pSTAT3 on the Tyr705 expression, and increases the autophagy [82]. 

Polarized macrophages also differ in terms of the cyto-/chemo-kines production, and M2 macrophages also exert their pro-tumoral activities by releasing specific cyto-/chemo-kines, which are involved in the key mechanisms in the tumor progression, such as angiogenesis [80]. Indeed, the M2 macrophages release IL-10, which in turn promotes VEGF production, and it has been demonstrated that rapamycin reduces angiogenesis by down-regulating the IL-10 and VEGF secretion [83].

mTORC2 also plays a critical role in M2 macrophages polarization; indeed, the inhibition of mTORC2 signaling in macrophages, by the deletion of rictor, reduces the differentiations of the M2 macrophages [84]. More recently, Shrivastava and colleagues demonstrated that mTORC2 up-regulates the M2 surface markers, CD206 and CD163, through the AKT axis, thus leading to an increase of the invasion and metastasis in mammary tumor models, both in in vitro and in mice [85].

### 4.3. MDSCs

MDSCs are a heterogeneous group of immature myeloid cells at various stages of differentiation, including precursors of macrophages, granulocytes, and dendritic cells (DC). Even if understanding the phenotypic and functional characteristics of the MDSCs is controversial, MDSCs can be classified in granulocyte or monocyte, based on the expression of Ly6C and Ly6G molecules, and play critical roles in primary and metastatic cancer progression. mTOR signaling is involved in the modulation of the MDSCs recruitment in TME, both in cancer cells and MDSCs; indeed, in cancer cells, the mTOR axis regulates the release of the soluble factors involved in the MDSCs recruitment, whereas in MDSCs, mTOR signaling affects the expression of specific antigen on their surface. 

Welte et al. demonstrated that mTOR signaling promotes MDSCs accumulation by up-regulating granulocyte colony-stimulating factor (G-CSF) in breast cancer cells. Indeed, both the rapamycin treatment and the deletion of Raptor reduce the G-CSF levels [86]. Another soluble factor involved in the recruitment of MDSCs in TME is TGF-β, a cytokine that directly promotes the expression of CD39^+^/CD73^+^ on the surface of myeloid cells, thereby exerting tumor-promoting roles [87]. Several studies in mouse models have demonstrated that the expression of these two ectonucleotidases leads to tumor cells’ evasion from cytotoxic T cells responses, and that mTOR plays a critical role in the regulation of the CD39/CD73 expression. Indeed, it has been reported that rapamycin abrogates the TGF-β-mediated induction of CD39/CD73 expression, by HIF-1α [50]. 

Despite the role of mTOR, described above, in enhancing pro-tumoral MDSCs accumulation, other groups demonstrate an opposite aspect of the MDSCs recruitment mTOR-mediated, highlighting the controversial function of the MDSCs in TME. Indeed, it has been demonstrated that the rapamycin treatment upregulates the recruitment and the induction of MDSCs’ immunosuppressive ability, by enhancing the production of IL-1 and IL-2, and by upregulating the expression of their effectors, arginase-1 and inducible nitric oxide synthase, which prevents T-cell proliferation [51]. 

Moreover, MDSCs can also affect tumor progression by modulating the commitment of the TME’ components. Indeed, preliminary in vitro studies indicate a potential role of MDSCs in mTOR-mediated CD8^+^ T cells differentiation into effector populations [88].

## 5. mTOR and Angiogenesis 

TME is composed not only of the cells of the immune system, but also of tumor vasculature, and angiogenesis has long been recognized as a hallmark of cancer [89]. Angiogenesis represents the mechanism by which new blood vessels develop, and it is a dynamic and tightly regulated process, mainly induced by hypoxia [27]. During the tumor progression, pathological angiogenesis is driven by the presence of pro-angiogenic factors in TME, such as VEGF-A and IL-8. Several cells, including tumor and tumor-associated stromal cells (such as endothelial cell and macrophages), are involved in this process, in part, by secreting growth factors and cytokines [27].

The VEGF/VEGF receptor (VEGFR) axis is one of the key regulators of angiogenesis, as demonstrated by the use of anti-VEGF/VEGFR drugs, to inhibit angiogenesis in cancer therapy, and it is regulated, among others, by the PI3K-AKT-mTOR signaling pathway [33,90]. Indeed, mTORC1 regulates the HIF-1/HIF-2, which are the transcription factors for the hypoxic stress response genes, including VEGF and TGF-α [6,91]. HIF-1, the predominant form, is a heterodimer consisting of HIF-1α and HIF-1β subunits; although HIF-1β is constitutively expressed, the stability of HIF-1α is dependent on oxygen levels [92]. In the presence of O_2_, the ubiquitination of HIF-1α induces its degradation by the 26S proteasome, whereas under hypoxic conditions, the accumulation of the HIF-1α subunit is because of a decrease in the rate of proteolytic degradation [92]. 

In addition to the HIF protein stability regulation, other mechanisms of the HIF-1α expression have been proposed at different levels, such as mRNA transcription and translation [93]. Recently, Dodd and colleagues demonstrated that mTORC1 enhances the transcription of HIF-1α mRNA, by directly phosphorylating STAT3 at Ser727 [94]. The same group also showed that mTORC1 regulates the HIF-1α translation, through the activity of both p70*^S6K1^* and elongation initiation factor (EIF)-4E binding protein 1 (4E-BP1) [94]. Conversely, several studies reported that long-lasting hypoxic conditions downregulate mTORC1 activity, by activating the negative mTORC1 regulators tuberous sclerosis complex TSC1–2 or 5′-AMP activated protein Kinase β (AMPK-β), which in turn phosphorylates Raptor on Ser722/792 [95,96]. 

This tight connection between the mTOR pathway and angiogenesis regulation is also maintained in endothelial cells, as demonstrated by the effects of the mTOR inhibitors. Indeed, several groups showed that rapamycin displays anti-angiogenic properties in terms of a reduction of the proliferation, migration, tubular structures formation and an increase of apoptosis [61,62,63]. Our previous data also demonstrated that the mTOR inhibitor temsirolimus inhibits serum- and/or VEGF-driven endothelial cell proliferation and vessel formation in vitro and in vivo [33]. Specific knock-down experiments of mTORC1/2 interactors in endothelial cells confirmed the role of mTOR in angiogenesis; indeed, knocking-down TSC1, a negative regulator of mTORC1, increases the proliferation of endothelial cells, whereas the deletion of rictor, a positive regulator of mTORC1, reduces cell proliferation [52,53]. 

As reviewed by De Palma and others, hematopoietic-derived tumor-infiltrating cells also regulate angiogenesis, by releasing growth factors and inflammatory cytokines in the TME [27]. Among them, TAMs play a critical role in tumor angiogenesis, because they exert a dual role, in both the inhibition and activation of angiogenesis [97]. Interestingly, even in TAMs, the mTOR activity displays a key role in promoting angiogenesis and polarization into the M1 phenotype [81]. Moreover, macrophages secrete pro-angiogenic factors such as VEGF-A, IL-1β, IL-8, and metalloproteases (MMP) 2 and 9, which are released also by other tumor infiltrating cells, such as neutrophils, eosinophils, natural killers, and CAFs [27]. O_2_ deprivation in specific tumor areas is a critical cue for the accumulation of TAMs, which are recruited by a hypoxia-induced chemoattractant gradient, such as VEGF, and endothelins [24].

Even if mTOR is a key regulator of HIF-1, the activation of other signaling pathways, which converge on the targets shared with the PI3K network, can also promote tumor angiogenesis. For example, the epidermal growth factor receptor (EGFR) activation increases the transcription of VEGF via the PI3K pathway, but not dependently on HIF-1, in glioblastoma cells; moreover, the EGFR amplification has an additive effect with a PTEN loss of function in increasing the VEGF levels, by enhancing the VEGF promoter activity [98,99]. Zundel and colleagues have demonstrated that in glioblastoma cell lines, a loss of PTEN results in HIF-1 stabilization which in turn causes the up-regulation of VEGF expression [100]. The loss of PTEN is a common mechanism of PI3K activation in several cancers, and its association with higher levels of secreted VEGF was observed also in other cancer cell lines with a different histological origin, such as pancreatic and prostate cancer cells, suggesting that PTEN plays a critical role in angiogenesis and tumorigenesis [101,102].

## 6. mTOR in CAFs Regulation

CAFs are cellular components of the stroma derived from the activated quiescent fibroblasts surrounding cancer cells, that directly promote tumor initiation, progression and metastasis by secreting growth factors, cytokines, and a large number of metabolites [103,104,105]. Moreover, the CAFs regulate normal epithelial differentiation and homeostasis, and cancer progression, particularly in stroma-rich tumors, like pancreatic cancers [106]. Indeed, in pancreatic ductal adenocarcinoma (PDAC), the stroma forms more than 80% of the tumor mass, and the CAFs express α-SMA (alpha-smooth muscle actin) and are also called activated pancreatic stellate cells [106]. Several studies demonstrated that CAFs display pro-tumorigenic properties by promoting invasion and metastasis in a non-vascular manner [107,108].

Similarly, recent evidence has showed that pancreatic CAFs may be involved in resistance to anticancer drugs [109,110]. Indeed, Duluc et al. demonstrated that, in pancreatic CAFs, the PI3K/mTOR pathway is activated by the autocrine secretion of PDGF and Janus kinase (JAK)2-dependent cytokines [54]. The CAFs also express the sst1 somatostatin receptor, whose activation inhibits the mTOR/4E-BP1 pathway, which in turn modulates the synthesis of the secreted proteins involved in the resistance to cancer drug therapies, including IL-6 and STAT3, and could be a possible upstream regulator of the IL-6 expression. These results suggest that counteracting the mTOR/IL-6 driven resistance might increase the effectiveness of the anticancer therapy [54,111]. Moreover, the specific inhibition of mTORC1 can also reverse the CAF-induced resistance through JAK/STAT3-, ERK- and AKT-signaling. Everolimus treatment, indeed, induces a significant reduction of the secretion of cytokines, such as IL-8, IL-13, and MCP-1, which are involved in the promotion of tumor cells’ proliferation and migration [64].

Wang et al. demonstrated that CAFs promoted irradiated cancer cell recovery through the increase of autophagy, thus causing a tumor relapse after radiation therapy. Indeed, they ensured that CAFs, through the production of IGF1/2, IL-12, and β-hydroxybutyrate, were capable of inducing autophagy in cancer cells post-radiation, thus increasing the level of reactive oxygen species (ROS), which in turn enhances protein phosphatase 2 (PP2)A activity, blocks mTORC1 activation, and induces autophagy in cancer cells [112].

## 7. Implications in Cancer Immunotherapy

In addition to the central role of the PI3K/AKT/mTOR signaling network dysregulation in cancer cells, recent evidence highlights that targeting this pathway can also impact on the host immunity [17]. In the past years, the host’s immune system has become a target for the development of new therapeutic strategies, such as immunomodulatory drugs or monoclonal antibodies, and cancer immunotherapy has achieved remarkable clinical efficacy in the treatment of many cancer patients, by promoting the antitumor activity of the immune system [4]. More specifically, two immune checkpoints have achieved the most attention so far, as follows: the cell surface protein PD-1 is expressed by activated T cells and the binding with its two ligands, PD-L1 and PD-L2, attenuates the activity of the T cells and the effector responses; CTLA-4 is a negative regulator of T cells, by competing with the co-stimulatory molecule CD28 for the binding to the ligands CD80 and CD86 [113]. The recruitment of regulatory immune cells can also mediate immune suppression; indeed, the modulation of T cell-mediated antitumor responses represents another therapeutic strategy [114]. Although blocking these checkpoints and the T cell-mediated immunotherapy exhibit clinical success, the majority of patients still fail to respond to immunotherapy, thus understanding the molecular mechanisms of resistance remains crucial to select a specific subset of patients and improve the overall survival [115].

As highlighted above, mTOR plays a central role in coordinating the environmental stimuli and cell metabolic responses, also because of its function in immune cell homeostasis and activation, and thus represents a potential target for cancer immunotherapy [116]. The first evidence of the tight connection between mTOR and immune regulatory targets was demonstrated by the immunosuppressive properties of the mTOR inhibitor rapamycin, because of its ability to block T cells proliferation [117]. In the last years, PD-L1 regulation has become the focus of many studies and several groups demonstrated that the PI3K/AKT/mTOR pathway regulates PD-L1 in different tumors, such as in non-small cell lung cancer [78]. As PI3K activation may occur via the loss of PTEN, it has been further reported that the loss of this tumor suppressor may induce the overexpression of PD-L1 and immunoresistance in several tumors of different histological origin (i.e., glioma, leiomyosarcoma, colorectal, breast cancer, and PDAC [118,119,120,121,122]. Mittendorf and her group also demonstrated that treatments with either the AKT inhibitor, MK-2206, or the mTOR inhibitor, rapamycin, significantly decreases the PD-L1 mRNA transcripts in the PTEN-mutant triple-negative breast cancer cell lines [121]. More recently, it has been also demonstrated that a loss of the PTEN expression correlates with a decrease of CD8^+^ T cells’ infiltration in melanoma and poor outcomes to immunotherapies, regardless of BRAF and NRAS status [56]. As opposed to PTEN, mTORC1 displays a controversial role in CD8^+^ T cells; indeed, the mTORC1 activity is required for their effector functions, and the inhibiting mTORC1 activity displays paradoxical immune stimulating effects by promoting memory CD8^+^ generation in a dose- and duration-dependent manner, as well [40].

CD8^+^ T cells are not the only regulators of the adaptive immune response involved in cancer immunotherapy; the dysregulation of CD4^+^ Treg cells are also involved in several pathological immune diseases and drug resistance. The mTOR and dual PI3K/mTOR inhibitors have been shown to induce Tregs’ expansion and their immunosuppressive activity, thus correlating with a poor prognosis in cancer patients [123]. Encouragingly, Huijts and her group showed that the PI3K inhibition alone allows for the expansion of Tregs, but without affecting their overall suppressive activity [124].

In addition to the upregulation of Treg into tumor tissues, mTOR signaling stimulates also the infiltration of MDSCs, thus allowing for it to combine immunotherapy with PI3K-AKT-mTOR pathway inhibitors. The activation of toll-like receptors (TLR) on DCs leads to their maturation, and the TLR signaling activates PI3K; this evidence highlights the synergistic effects of the combined PI3K inhibitors with TLR agonists in mouse models [125]. 

## 8. Conclusions

The PI3K/AKT/mTOR network plays a significant role in the regulation of several processes in tumor initiation and progression, by controlling the protein synthesis, proliferation, growth, and survival in cancer cells on the one hand, and by affecting the characterization and the activity of the TME’s elements on the other hand. The involvement of TME in cancer progression and drug resistance is well recognized, thus leading to a necessity to better investigate the molecular mechanisms of tumor-stroma interactions (TSI). Thus, targeting the molecular mediators of TSIs, such the elements of the mTOR network, may provide an excellent strategy for therapeutic opportunities.

## Figures and Tables

**Table 1:** Role of mammalian target of rapamycin (mTOR) complexes in tumor microenvironment (TME) elements. TAM—tumor-associated macrophages (TAM); MDSC—myeloid-derived suppressor cells; CAF—cancer-associated fibroblasts; IL—interleukin; mTORC1—mTOR complex 1; ↓ indicates a decrease of activity; ↑ indicates an increase of activity.

Element of TME.	mTORC1/2 Modulation	Effects of Modulation	References
CD8^+^	↓ mTORC1	↓ Effector ↑ Memory	[39,40]
	↓ mTORC2	↓ Memory	[39]
CD4^+^	↑ mTORC1/2	↑ Th1, 2, 17 differentiation	[41,42,43]
Treg	↑ mTORC1	↑ Differentiation in effector-like T cells	[44]
	↑ mTORC2	↓ Differentiation	[45,46]
TAM	↑ mTORC1/2	↑ M2 polarization	[47,48]
MDSC	↓↑ mTORC1	Variable effects	[49,50,51]
Endothelial cells	↑ mTORC1	↑ Proliferation	[52,53]
CAF	↓ mTORC1	↓ IL-6 secretion	[54]

**Table 2:** mTOR-axis inhibitors and potential therapeutic benefit. PI3K—phosphoinositide 3-kinase; Treg—regulatory T cells.

Drug(s)	Target Cell Population	Functional Implication	Potential Therapeutic Benefit	References
mTOR/p110β/pan-PI3K inhibitors	CD8^+^	↑ CD8^+^ infiltration in tumor	↑ Significant survival benefit	[39,56,57]
mTORC1 inhibitor	CD4^+^	↓ number of CD4^+^	↑ Significant survival benefit	[58]
mTOR/pan-AKT inhibitors	Treg	↓ Tregs selectively	↑ Significant survival benefit	[58,59]
PI3K inhibitors	TAM	↓ TAM recruitment	Variable effects	[60]
mTOR inhibitors	MDSC	Variable effects	Variable effects	[49,51]
mTOR inhibitors	Endothelial cells	↓ proliferation, migration and tubular structures formation ↑ apoptosis	↓Angiogenesis	[33,61,62,63]
mTORC1 inhibitor	CAF	↓ CAF-secreted cytokines	↓ Of cell migration, invasion, and metastasis	[64]

## Abbreviations

AMPK-β	5′-AMP activated protein kinase β
CAF	cancer-associated fibroblasts
CTLA-4	cytotoxic t-lymphocyte protein 4
DC	dendridic cell
DEPTOR	DEP domain-containing mTOR interacting protein
ECM	extracellular matrix
EGFR	epidermal growth factor receptor
FOXO	forkhead box
G-CSF	granulocyte colony-stimulating factor
HIF	hypoxia-inducible factor
Hsp70	heat shock protein 70
IGF	insulin growth factor
IL	interleukin
JAK	Janus kinase
MDSC	myeloid-derived suppressor cells
mLST8	mammalian lethal with sec13 protein 8
MMP	metalloproteases
mSIN1	mammalian stress-activated protein kinase interacting protein 1
mTOR	mammalian target of rapamycin
mTORC	mTOR complex
PD-1	programmed death 1
PDAC	pancreatic ductal adenocarcinoma
PDGF	plated-derived growth factor
PDK1	phosphoinositide-dependent kinase-1
PIP2	phosphatidylinositol 4,5-bisphosphate
PIP3	phosphatidylinositol 3,4,5-trisphosphate
PI3K	phosphoinositide 3-kinase
PKC	protein kinase C
PP2	protein phosphatase 2
PRAS40	proline-rich AKT substrate 40
PRR	proline-rich protein
Protor	protein observed with rictor
PTEN	phosphatase and tensin homolog on chromosome 10
p70^S6K1^	p70 ribosomal protein S6 kinase 1
ROS	reactive oxygen species
SGK	serum and glucocorticoid kinase
SREBP	sterol regulatory element binding proteins
STAT	signal transducer and activator of transcription
TAM	tumor-associated macrophages
TCR	T cell receptor
Tel2	telomere maintenance 2
TGF	trasforming growth factor
Th	T helper
TLR	tool-like receptor
TME	tumor microenvironment
Treg	regulatory T cells
TSC	tuberous sclerosis complexes
TSI	tumor-stroma interactions
Tti1	tel2-interacting protein 1
VEGF	vascular endothelial growth factor
VEGFR	VEGF receptor
4EBP-1	4E binding protein-1
